# Twenty‐Year Kidney Transplant Outcomes With Prednisone‐Free Maintenance Immunosuppression: A Matched Control Analysis

**DOI:** 10.1111/ctr.70297

**Published:** 2025-10-14

**Authors:** Lindsey Turner, David M. Vock, Erika Helgeson, Raja Kandaswamy, Richard Spong, Timothy Pruett, Erik Finger, Vanessa Humphreville, Srinath Chinnakotla, Andrew Adams, Rasha el‐Rifai, Karthik Ramanathan, Arthur J. Matas

**Affiliations:** ^1^ Division of Biostatistics and Health Data Science School of Public Health University of Minnesota Minneapolis Minnesota USA; ^2^ Division of Transplantation Department of Surgery University of Minnesota Minneapolis Minnesota USA; ^3^ Division of Nephrology Department of Medicine University of Minnesota Minneapolis Minnesota USA

**Keywords:** donors and donation, immunosuppressant, kidney transplantation

## Abstract

There has been concern about long‐term outcomes of kidney transplant recipients treated with prednisone‐free maintenance immunosuppression. We studied 20‐year outcomes for recipients treated with discontinuation of prednisone <1 week posttransplant (rapid discontinuation of prednisone [RDP]) compared with contemporaneous matched controls treated with maintenance prednisone (MP). First and second, adult living donor (LD) and deceased donor (DD) kidney transplant recipients from 1999 to 2009, treated with RDP were matched—using data from the Scientific Registry of Transplant Recipients—with contemporaneous controls, from large transplant centers, who were treated with MP. A total of 361 DD recipients treated with RDP were matched with 1805 treated with long‐term MP. A total of 763 LD recipients treated with RDP were matched with 2289 treated with long‐term MP. DD recipients treated with RDP had significantly better recipient survival (*p* = 0.02); there was no difference in graft or death‐censored graft survival. For LD recipients, there was no difference between groups for all outcomes. Kidney transplant recipients treated with RDP had better or similar long‐term outcomes as those treated with long‐term MP. RDP should be considered for the majority of kidney transplant recipients.

**Abbreviations**
BMI: body mass index
CI: confidence interval
DD: deceased donor
HLA: human leukocyte antigen
HR: hazard ratio
LD: living donor
MP: maintenance prednisone
PRA: panel‐reactive antibody
RDP: rapid discontinuation of prednisone
SMD: standardized mean difference
SRTR: Scientific Registry of Transplant Recipients
UNOS: United Network for Organ Sharing
WBC: white blood cell

## 1 Introduction

Before 2000, kidney transplant immunosuppression in the United States routinely included high‐dose prednisone (up to 2 mg/kg/day) followed by a slow taper over the first posttransplant year. Not surprisingly, prednisone‐related complications (e.g., cataracts, new‐onset diabetes, avascular necrosis of the hip, fractures, hyperlipidemia, weight gain, skin changes, and cushingoid appearance, and in children, growth retardation) were common [1, 2]. When asked, transplant recipients stated that prednisone was the drug they would most want eliminated from their immunosuppressive protocol [3]. Consequently, there were numerous attempts to develop prednisone minimization protocols. In randomized trials of recipients treated with prednisone, a calcineurin inhibitor and either azathioprine or mycophenolate, late prednisone withdrawal ≥3 months posttransplant lead to increased acute rejection rates and increased graft loss [4, 5, 6]. In contrast, numerous short‐ and medium‐term randomized studies have shown that protocols incorporating rapid discontinuation of prednisone (RDP) (prednisone stopped in the first two posttransplant weeks), was associated with similar patient and graft survival as protocols incorporating long‐term maintenance prednisone (MP) [7, 8, 9, 10, 11, 12, 13, 14, 15, 16, 17, 18, 19, 20, 21, 22, 23].

In spite of the benefits of RDP—that is, minimization of prednisone‐related complications, without decreasing patient and graft survival—in the United States, only 30%–35% of new kidney transplant recipients each year are started on a protocol incorporating RDP [24], in part due to concern about late outcomes [25]. To address this concern, using data from the University of Minnesota kidney transplant recipient database and the Scientific Registry of Transplant Recipients (SRTR), we compared 20‐year patient and graft survival of recipients treated with RDP to contemporaneous matched recipients treated with long‐term MP.

## 2 Methods

### 2.1 Study Population

The RDP protocol at the University of Minnesota has previously been described [26]. Methylprednisolone was given intraoperatively; oral prednisone was started on postoperative Day 1, tapered over 5 days, and discontinued on postoperative Day 6. The dosing of other immunosuppressive agents—antithymocyte globulin, mycophenolate, and a calcineurin inhibitor—was unchanged from previous protocols incorporating long‐term MP. After a pilot trial in first living donor (LD) transplants [27], RDP was used for all first and second LD and deceased donor (DD) transplants, with the exception of recipients already taking prednisone at the time of transplant. In order to focus on long‐term outcomes and minimize era effects, we studied outcomes of adult (18 years of age or older) RDP‐treated kidney‐alone recipients with a transplant between September 30, 1999 (when the clinical RDP protocol was initiated at our center), and September 30, 2009. Recipient data is maintained in an IRB‐approved database.

RDP‐treated transplant recipients at the University of Minnesota were compared—using data from the SRTR—with kidney‐alone, MP‐treated matched controls from centers doing >500 transplants during the 10‐year study interval. We took an intention‐to‐treat approach and categorized patients as either on RDP or MP based on whether or not steroids were part of the maintenance immunosuppression on the United Network for Organ Sharing (UNOS) transplant registration form, which is completed at hospital discharge.

### 2.2 Statistical Analysis

Recipients undergoing RDP (*n* = 1124 transplants: 361 DD; 763 LD) were matched *k*:1 to MP‐treated recipients. We used nearest‐neighbor propensity score matching with exact matching on donor type (DD vs. LD) and transplant number (first vs. second transplant). In the matching, *k* was chosen to be as large as possible so that sufficient balance (standardized mean difference [SMD] < 0.1) was achieved for all covariates included in the propensity score. Logistic regression was used to estimate the propensity score for undergoing RDP. We fit separate propensity score models for LD and DD recipients. The propensity score model for LDs included relevant recipient characteristics (age at transplant, race [Black/non‐Black], sex, body mass index (BMI), functional status, pre‐emptive transplant, primary disease, diabetes mellitus, peak panel‐reactive antibody [PRA], induction immunosuppression, and transplant year), donor characteristics (age, race [Black/non‐Black], sex, hypertension, creatinine), and recipient/donor matching (number of human leukocyte antigen [HLA] mismatches). The propensity score model for DDs additionally included cold ischemic time and donor cause of death. We used multivariate imputation by chained equations to impute the missing data by predictive mean matching for the variables that were used in the matching algorithm [28].

To assess the effect of steroid group (RDP vs. MP) on patient survival, graft survival, and death‐censored graft survival, we fit proportional hazards models. These models were used to estimate adjusted survival probability at 20 years. Separate models were fit for LD and DD recipients. As a sensitivity analysis, first and second transplants were analyzed separately. During the studied interval, our center switched from cyclosporine‐based to tacrolimus‐based immunosuppression. As a consequence, we were unable to match on a maintenance immunosuppression regimen, which remained imbalanced among the groups. We, therefore, adjusted for maintenance immunosuppression. As recommended by Stuart [29], we also included in the proportional hazards models those covariates that we matched on to improve efficiency and reduce any residual confounding from covariates that remain imbalanced after matching.

Follow‐up began at hospital discharge and was censored at the last follow‐up. All statistical analyses were performed using R version 4.1.2 (R Foundation for Statistical Computing, Vienna, Austria). *p* values < 0.05 were considered statistically significant.

## 3 Results

### 3.1 Deceased Donor Recipients

Between September 30, 1999, and September 30, 2009, there were 361 DD kidney transplant recipients at the University of Minnesota that met the inclusion and exclusion criteria and were treated with RDP. Of the 361, 297 were first transplant recipients;, 64 second. Median (quartiles) follow‐up was 10.0 (5.6, 14.1) years. Each of the RDP patients was matched to five MP‐treated patients from other centers (1805 MP were matched) (Table 1). In general, after matching, characteristics on which we matched were well‐balanced between groups with SMD < 0.1 for all matched characteristics (Table 1).

**TABLE 1:** Donor and recipient characteristics of those treated with rapid discontinuation of prednisone (RDP) compared with maintenance prednisone (MP) among deceased donor recipients.

	Level	MP	RDP	SMD
*N*		1805	361	
Recipient age at transplant (years) (IQR)		52.2 [41.9, 61.0]	54.2 [40.5, 62.9]	0.010
Recipient race	Not Black or African American	1668 (92.4)	334 (92.5)	0.004
	Black or African American	137 (7.6)	27 (7.5)	
Recipient sex	Female	827 (45.8)	165 (45.7)	0.002
	Male	978 (54.2)	196 (54.3)	
Recipient primary disease	Type 1 diabetes	183 (10.1)	43 (11.9)	0.080
	Type 2 diabetes	330 (18.3)	61 (16.9)	
	Focal segmental glomerulosclerosis	102 (5.7)	22 (6.1)	
	Hypertension	218 (12.1)	45 (12.5)	
	IgA nephropathy	92 (5.1)	15 (4.2)	
	Other	704 (39.0)	139 (38.5)	
	Polycystic kidney disease	176 (9.8)	36 (10.0)	
Transplant year		2006.5 [2004.0, 2008.4]	2006.5 [2004.3, 2008.2]	0.028
Donor age (years)		42.0 [25.0, 52.0]	41.0 [23.0, 52.0]	0.020
Donor race	Not Black or African American	1722 (95.4)	341 (94.5)	0.043
	Black or African American	83 (4.6)	20 (5.5)	
Most recent peak % PRA		2.0 [0.0, 50.0]	2.0 [0.0, 51.0]	0.010
Donor sex	Female	711 (39.4)	141 (39.1)	0.007
	Male	1094 (60.6)	220 (60.9)	
Induction immunosuppression	Campath	12 (0.7)	4 (1.1)	0.059
	Interleukin‐2	20 (1.1)	3 (0.8)	
	None	31 (1.7)	6 (1.7)	
	Other induction regimen	88 (4.9)	16 (4.4)	
	Thymoglobulin	1654 (91.6)	332 (92.0)	
Number of HLA mismatches	0	395 (21.9)	84 (23.3)	0.066
	1	21 (1.2)	4 (1.1)	
	2	133 (7.4)	29 (8.0)	
	3	250 (13.9)	54 (15.0)	
	4	405 (22.4)	76 (21.1)	
	5	388 (21.5)	75 (20.8)	
	6	213 (11.8)	39 (10.8)	
Received previous kidney	No	1485 (82.3)	297 (82.3)	<0.001
	Yes	320 (17.7)	64 (17.7)	
Recipient diabetes diagnosis	No	1214 (67.3)	243 (67.3)	0.001
	Yes	591 (32.7)	118 (32.7)	
Pre‐emptive transplant	No	1490 (82.5)	297 (82.3)	0.007
	Yes	315 (17.5)	64 (17.7)	
Recipient BMI (mg/m^2^)		27.0 [23.4, 31.3]	27.3 [23.1, 31.8]	0.017
Recipient functional status	No Assistance	1377 (76.3)	275 (76.2)	0.030
	Some Assistance	409 (22.7)	81 (22.4)	
	Total Assistance	19 (1.1)	5 (1.4)	
Donor hypertension	No	1459 (80.8)	293 (81.2)	0.008
	Yes	346 (19.2)	68 (18.8)	
Donor creatinine (mg/dL)		0.9 [0.7, 1.2]	0.9 [0.7, 1.2]	0.020
Cold ischemic time (h)		14.5 [9.5, 20.0]	14.2 [10.2, 19.0]	0.005
Donor cause of death	Other	833 (46.1)	167 (46.3)	0.026
	Anoxia	295 (16.3)	62 (17.2)	
	Stroke	677 (37.5)	132 (36.6)	
Maintenance immunosuppression	Cyclosporine mycophenolate mofetil	167 (9.3)	234 (64.8)	1.616
	Other maintenance regimen	280 (15.5)	20 (5.5)	
	Tacrolimus mycophenolate mofetil	1328 (73.6)	78 (21.6)	
	Tacrolimus mTOR	30 (1.7)	29 (8.0)	

*Note:* Continuous variables were summarized by median [interquartile range], and categorical variables were summarized by frequency (percent).

Abbreviations: BMI, body mass index; HLA, human leukocyte antigen; PRA, panel‐reactive antibody; SMD, standardized mean difference.

**FIGURE 1:**
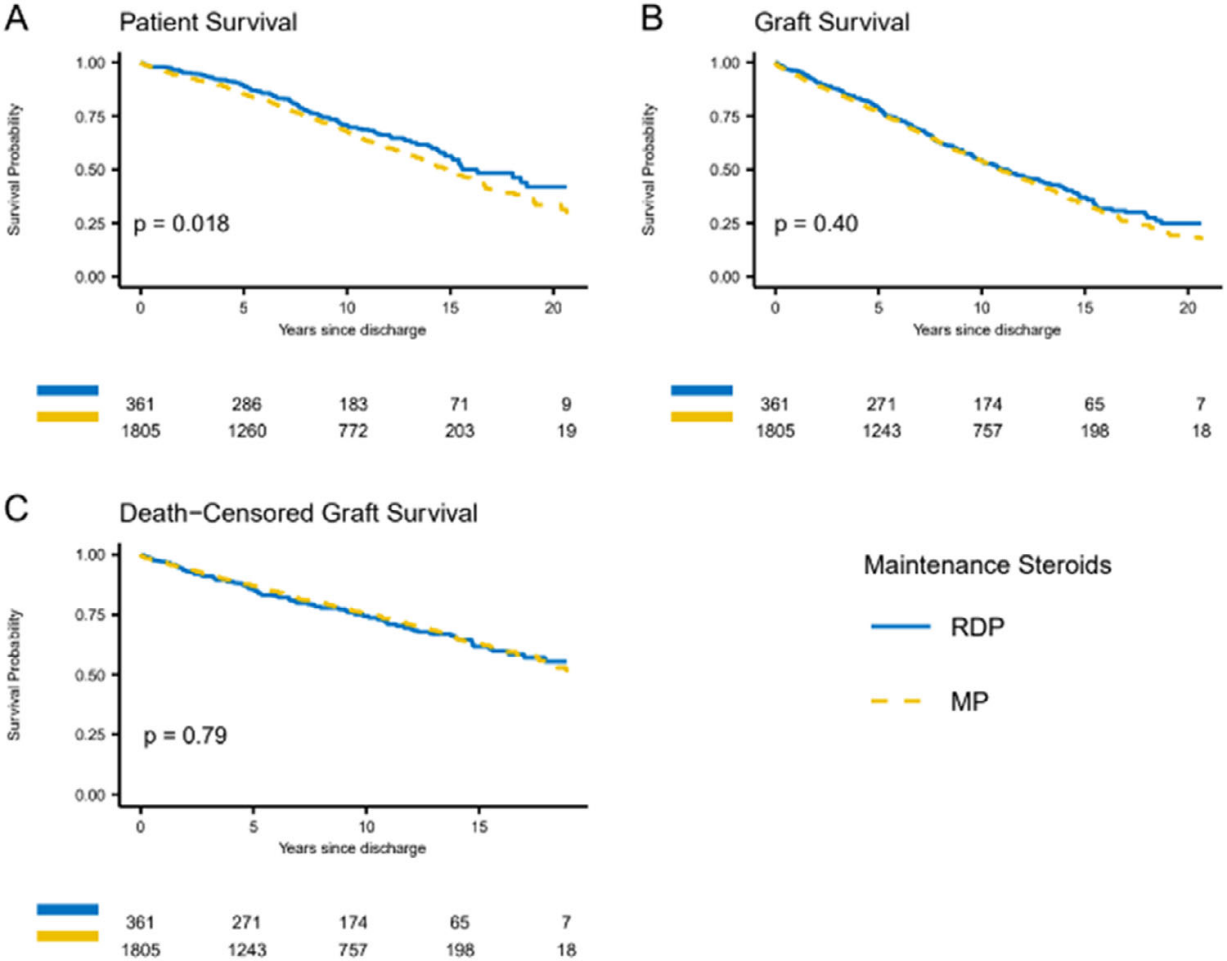
Adjusted patient (A), graft (B), and death‐censored graft (C) survival for deceased donor transplants treated with rapid discontinuation of prednisone (RDP) compared with maintenance prednisone (MP).

Patient survival was significantly better for the RDP group (hazard ratio [HR] = 0.76, 95% confidence interval [CI]: 0.61–0.95, *p* = 0.02 [Table 2]), with an estimated 20‐year patient survival for RDP of 39.0%; for MP, 32.3% (Figure 1). There was no significant difference in graft survival (HR = 0.93, 95% CI: 0.78–1.11, *p* = 0.40) and death‐censored graft survival (HR = 1.04, 95% CI: 0.80–1.34, *p* = 0.79) between the two groups (Table 2, Figure 1). The estimated 20‐year graft survival for RDP was 21.0%; for MP, 19.0%; the estimated 20‐year death‐censored graft survival for RDP was 51.2%; for MP, 52.3% (Figure 1). Of the patients on the RDP protocol, 90.8% were steroid‐free at 6 months and 81.4% were steroid‐free at 12 months.

**TABLE 2:** Adjusted hazard ratios comparing rapid discontinuation of prednisone (RDP) to maintenance prednisone (MP) for death‐censored graft survival, graft survival, and patient survival.

	HR (95% CI), *p* value	
Death‐censored graft survival	Living donor	Deceased donor
Overall	0.99 (0.82, 1.21), *p* = 0.94	1.04 (0.80, 1.34), *p* = 0.79
First transplant	0.99 (0.80, 1.22), *p* = 0.91	0.97 (0.73, 1.30), *p* = 0.85
Second transplant	1.14 (0.59, 2.20), *p* = 0.70	1.24 (0.68, 2.26), *p* = 0.49
Graft survival		
Overall	0.95 (0.82, 1.10), *p* = 0.50	0.93 (0.78, 1.11), *p* = 0.40
First transplant	0.96 (0.82, 1.12), *p* = 0.62	0.91 (0.75, 1.10), *p* = 0.32
Second transplant	0.97 (0.58, 1.64), *p* = 0.92	1.01 (0.63, 1.62), *p* = 0.97
Patient survival		
Overall	0.91 (0.75, 1.11), *p* = 0.37	0.76 (0.61, 0.95), *p* = 0.018
First transplant	0.94 (0.77, 1.16), *p* = 0.59	0.77 (0.61, 0.99), *p* = 0.039
Second transplant	0.83 (0.38, 1.83), *p* = 0.64	0.64 (0.32, 1.28), *p* = 0.20

*Note:* Hazard ratios (HRs) < 1 indicate a survival benefit for RDP.

For first transplants (Table S1), RDP was associated with significantly better patient survival (HR = 0.77, 95% CI: 0.61–0.99, *p* = 0.04) (Figure S1). There was no difference between groups in graft or death‐censored graft survival (*p* = 0.32 and 0.85, respectively). For second transplants (Table S2), there was no statistically significant difference between the RDP group and MP group for overall patient (*p* = 0.20), graft (*p* = 0.97), and death‐censored graft survival (*p* = 0.49), although the number of DD second transplant recipients is small and the point estimates for the HR comparing RDP to MP is similar between first and second DD transplant (Figure S2).

### 3.2 Living Donor Recipients

Between September 30, 1999, and September 30, 2009, there were 763 LD recipients at the University of Minnesota that met the inclusion and exclusion criteria and were treated with RDP. Of the 763, 698 were first transplant recipients; 65 second. Median (quartiles) follow‐up was 12.6 (7.6, 15.1) years. Each of the 763 RDP patients was matched to three MP patients (2289 were matched) (Table 3). However, primary kidney disease differed between the groups with the primary kidney disease more likely to be Type 1 diabetes for RDP (21.8%) than for MP (10.9%).

**TABLE 3:** Donor and recipient characteristics of those treated with rapid discontinuation of prednisone (RDP) compared with maintenance prednisone (MP) among living donor recipients.

	Level	MP	RDP	SMD
*N*		2289	763	
Recipient age at transplant (years) (IQR)		46.5 [34.3, 56.1]	46.8 [34.8, 55.5]	0.049
Recipient race	Not Black or African American	2214 (96.7)	737 (96.6)	0.007
	Black or African American	75 (3.3)	26 (3.4)	
Recipient sex	Female	848 (37.0)	280 (36.7)	0.007
	Male	1441 (63.0)	483 (63.3)	
Recipient primary disease	Type 1 diabetes	250 (10.9)	166 (21.8)	0.298
	Type 2 diabetes	259 (11.3)	72 (9.4)	
	Focal segmental glomerulosclerosis	171 (7.5)	49 (6.4)	
	Hypertension	206 (9.0)	59 (7.7)	
	IgA nephropathy	193 (8.4)	62 (8.1)	
	Other	891 (38.9)	260 (34.1)	
	Polycystic kidney disease	319 (13.9)	95 (12.5)	
Transplant year		2005.5 [2003.3, 2007.9]	2005.7 [2003.7, 2007.5]	0.032
Donor age (years)		41.0 [33.0, 49.0]	43.0 [34.0, 49.0]	0.017
Donor race	Not Black or African American	2218 (96.9)	741 (97.1)	0.013
	Black or African American	71 (3.1)	22 (2.9)	
Most recent peak PRA		0.0 [0.0, 7.0]	0.0 [0.0, 4.0]	0.021
Donor sex	Female	1380 (60.3)	465 (60.9)	0.013
	Male	909 (39.7)	298 (39.1)	
Induction immunosuppression	Campath	2 (0.1)	1 (0.1)	0.019
	Interleukin‐2	10 (0.4)	3 (0.4)	
	None	118 (5.2)	40 (5.2)	
	Other induction regimen	50 (2.2)	18 (2.4)	
	Thymoglobulin	2109 (92.1)	701 (91.9)	
Number of HLA mismatches	0	179 (7.8)	72 (9.4)	0.060
	1	119 (5.2)	38 (5.0)	
	2	396 (17.3)	127 (16.6)	
	3	642 (28.0)	214 (28.0)	
	4	328 (14.3)	106 (13.9)	
	5	412 (18.0)	137 (18.0)	
	6	213 (9.3)	69 (9.0)	
Received previous kidney	No	2094 (91.5)	698 (91.5)	<0.001
	Yes	195 (8.5)	65 (8.5)	
Recipient diabetes diagnosis	No	1716 (75.0)	507 (66.4)	0.188
	Yes	573 (25.0)	256 (33.6)	
Pre‐emptive transplant	No	1381 (60.3)	438 (57.4)	0.060
	Yes	908 (39.7)	325 (42.6)	
Recipient BMI (kg/m^2^)		25.8 [22.4, 30.0]	25.8 [22.2, 30.0]	0.043
Recipient functional status	No assistance	1971 (86.1)	658 (86.2)	0.032
	Some assistance	303 (13.2)	98 (12.8)	
	Total assistance	15 (0.7)	7 (0.9)	
Donor hypertension	No	2264 (98.9)	740 (97.0)	0.136
	Yes	25 (1.1)	23 (3.0)	
Donor creatinine (mg/dL)		0.9 [0.7, 1.0]	0.9 [0.8, 1.0]	0.038
Maintenance immunosuppression	Cyclosporine mycophenolate mofetil	234 (10.2)	465 (60.9)	1.575
	Other maintenance regimen	415 (18.1)	31 (4.1)	
	Tacrolimus mycophenolate mofetil	1598 (69.8)	174 (22.8)	
	Tacrolimus mTOR	42 (1.8)	93 (12.2)	

*Note:* Continuous variables were summarized by median [interquartile range], and categorical variables were summarized by frequency (percent).

Abbreviations: BMI, body mass index; HLA, human leukocyte antigen; PRA, panel‐reactive antibody; SMD, standardized mean difference.

**FIGURE 2:**
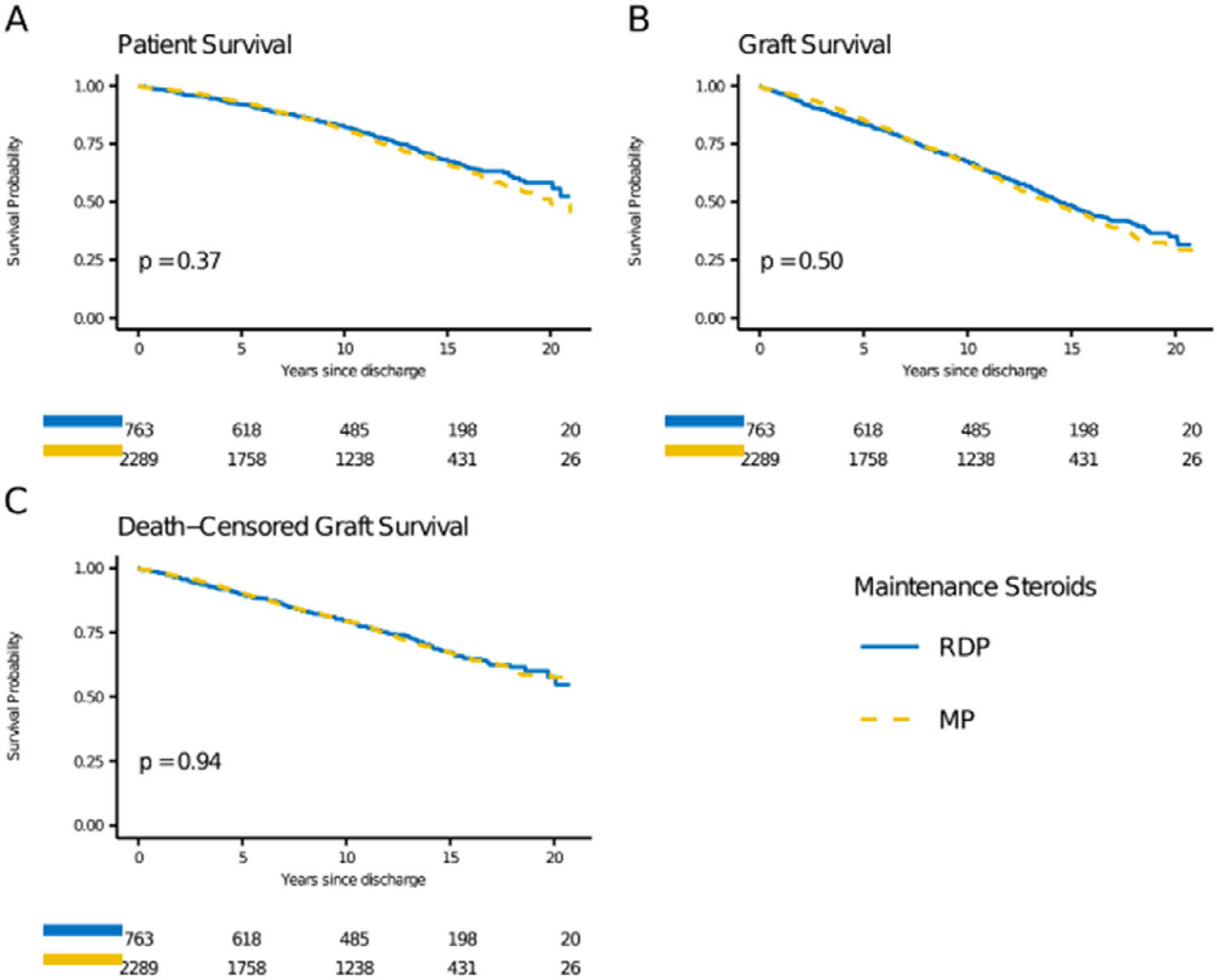
Adjusted patient (A), graft (B), and death‐censored gr aft (C) survival for living donor transplants treated with rapid discontinuation of prednisone (RDP) compared with maintenance prednisone (MP).

Patient survival was not significantly different between the RDP and MP groups (HR = 0.91, 95%CI: 0.75–1.11, *p* = 0.37; Table 2); the estimated 20‐year patient survival for RDP was 54.1% and for MP was 51.9% (Figure 2). There was also no significant difference between groups for graft survival (HR = 0.95, 95% CI: 0.82–1.10, *p* = 0.50) and death‐censored graft survival (HR = 0.99, 95% CI: 0.82–1.21, *p* = 0.94). The estimated 20‐year graft survival for RDP was 32.4% and for MP was 30.8%; the estimated 20‐year death‐censored graft survival for RDP was 56.7% and for MP was 56.5% (Figure 2). Of the RDP patients, 88.2% were steroid‐free at 6 months and 80.7% were steroid‐free at 12 months.

In separate analyses of the impact of RDP on first and second LD transplants (Tables S3 and S4), there was no statistically significant difference between the RDP and MP groups for overall patient, graft, or death‐censored graft survival (Figures S3 and S4).

## 4 Discussion

The goal of prednisone minimization protocols has been to minimize prednisone‐related side effects, without reducing patient and graft survival. Numerous randomized studies, reviews, and meta‐analyses have uniformly reported that, with RDP, these goals have been accomplished [7, 8, 9, 10, 11, 12, 13, 14, 15, 16, 17, 18, 19, 20, 21, 22, 23]. Yet the use of RDP in the United States has plateaued at 30%–35% of new recipients each year [24]. Additionally, RDP use differs markedly between centers, with some using it for almost all recipients, some using it selectively, and some always using long‐term MP [30]. It is not clear what leads to these differences.

When considering RDP for a population, or for a specific individual, a number of related concerns need to be addressed. The first is harm. Historically in kidney transplantation, acute rejection episodes have been associated with decreased graft survival. In some randomized RDP studies, but not others, RDP has been associated with an increased risk of early acute rejection episodes [7, 8, 9, 10, 11, 12, 13, 14, 15, 16, 17, 18, 19, 20, 21]. In general, these episodes have been easy to treat, and there has been no difference between groups in steroid‐resistant rejection. These same studies have shown no difference between groups in recipient survival, graft survival, death‐censored graft survival, or kidney function [7, 8, 9, 10, 11, 12, 13, 14, 15, 16, 17, 18, 19, 20, 21]. A limitation of these studies is that the majority have relatively short follow‐up. We previously compared RDP to *historical* MP‐treated controls at our center and reported that there was no difference between groups in 15‐year patient and graft survival [31]. In a 15‐year follow‐up of their randomized study, using national registry data, Woodle et al. similarly found no difference in outcomes between those randomized to RDP compared with MP [32]. To emphasize long‐term outcomes, our current analyses focused on recipients transplanted between 1999 and 2009. We found that RDP‐treated DD transplants, compared to matched, *contemporaneous*, MP‐treated controls, had significantly better 20‐year recipient survival (*p* = 0.02); there was no difference between groups in graft or death‐censored graft survival. For LD recipients, there was no difference between groups in 20‐year recipient, graft, or death‐censored graft survival.

Another limitation of randomized RDP studies is that enrollment has been restricted to low‐risk groups (e.g., first transplant, low PRA). However, multiple center and registry analyses have shown that for first kidney transplants in a number of “high risk recipients” [33, 34, 35, 36, 37, 38, 39, 40, 41, 42, 43, 44, 45, 46, 47, 48, 49, 50, 51, 52, 53, 54, 55, 56, 57, 58, 59, 60, 61, 62, 63, 64, 65, 66, 67, 68, 69]—that is, African Americans [33, 34, 35, 36, 37, 38, 39, 40], obese recipients [41], those with a potentially recurrent disease [34, 42, 43, 44, 45, 46, 47], pediatric recipients [34, 48, 49, 50, 51, 52, 53, 54, 55, 56, 57, 58, 59], recipients with HIV [60], recipients of expanded criteria donor kidneys [61], and simultaneous kidney/pancreas transplants [62, 63]—RDP, compared with MP, is associated with better or equivalent patient and graft survival (Table 4). Similarly, RDP is associated with equivalent outcomes in second LD transplants [34]. There are conflicting results for second DD transplants [34, 64, 65], sensitized recipients [34, 37, 66, 67], and those with delayed graft function (Table 4) [68, 69].

**TABLE 4:** Outcomes of Center and Registry Studies of RDP versus MP in “high risk” groups.

Recipient group	References
**A. No difference between RDP and MP in recipient and graft survival**	
1^st^ transplant	
African Americans	[33, 34, 35, 36, 37, 38, 39, 40]
Overweight recipients	[41]
Potentially recurring disease	[34, 42, 43, 44, 45, 46, 47]
Pediatric recipients	[34, 48, 49, 50, 51, 52, 53, 54, 55, 56, 57, 58, 59]
Recipients with HIV	[60]
Recipients of ECD kidneys	[61]
Simultaneous kidney/pancreas	[62, 63]
2^nd^ living donor recipients	[34]
**B. Conflicting data on RDP versus MP**	
2^nd^ deceased donor transplants	
No difference between groups	[34, 37, 64]
Decreased survival with RDP	[65]
Sensitized recipients	
No difference between groups	[34, 37, 66]
Decreased survival with RDP	[67]
Recipients with DGF	
No difference between groups	[68]
Decreased survival with RDP	[69]

A second consideration in the use of RDP is the benefit. Intuitively, if outcomes of RDP and MP protocols are similar, avoiding prednisone and the associated prednisone‐related side effects would be a reasonable approach. Clearly, compared to the high doses of prednisone used 2–3 decades ago, RDP is associated with a marked reduction in adverse events [70]. Today, most MP protocols use much lower prednisone doses, leading to the question of whether these low‐dose protocols are associated with prednisone‐related side effects. It is unlikely that there will ever be an adequately powered randomized trial to answer this question. However, in the general population, chronic low‐dose prednisone is associated with adverse events (e.g., loss of bone mineral density [BMD], increased risk of fractures) [71, 72, 73, 74, 75, 76, 77, 78, 79, 80, 81, 82]. Similarly, studies of recent cohorts of transplant recipients have reported that RDP is associated with improved BMD and decreased fracture risk compared to MP [83, 84, 85, 86, 87, 88]. Some, but not all, randomized studies have found RDP is associated with decreased risk of NODM and hyperlipidemia. Registry studies report both decreased NODM and reduced fracture risk with RDP compared to MP [89, 90, 91, 92]; a caveat is that there is no record of what dose of prednisone was used in the MP population.

There are other possible reasons that there may be hesitancy to use RDP. One concern may be that, with RDP, there is a need to use increased doses/blood levels of other maintenance immunosuppressive drugs. Yet, successful RDP protocols have not used increased doses or blood levels of other maintenance drugs. The potential for increased costs may limit RDP use. Successful RDP protocols have required antibody induction (thymoglobulin, alemtuzumab, or IL‐2r) [22, 23, 92]. However, in the United States today, over 90% of adult transplant recipients already receive antibody induction [24]. In addition, studies have shown that, because of the cost of treating increased side effects, MP protocols are associated with increased costs [88, 94, 95].

Finally, physicians who have extensive experience with MP may be uncomfortable initiating RDP protocols. RDP is associated with low white blood counts, and programs need to develop experience and understanding that with these low counts, recipients can still respond normally to infections. As described above, there is now a large body of evidence that for the vast majority of transplant recipients, RDP and MP protocols have equivalent patient, graft, and death‐centered graft survival.

Our study has limitations. This is a retrospective analysis of kidney transplant recipients from a single center compared to matched contemporaneous recipients in the SRTR. Our analysis is based on recipients starting an RDP protocol (intention‐to‐treat). Importantly, not all recipients tolerate RDP. In our series, approximately 20% were started on 5 mg prednisone daily within the first year posttransplant for reasons including low white blood cell (WBC), nontransplant‐related reasons, or following treatment of an acute rejection episode [96]. Most of our population is white, and we do not have sufficient recipients in this cohort to study high risk subgroups. But, as noted above, previous analyses of SRTR and other large databases have shown that most “high risk” groups (e.g., African Americans, potentially recurring disease) have similar outcomes with RDP and MP. Our SRTR data analyses were based on data provided on the UNOS registration form, which is completed at hospital discharge. It is possible that some recipients discharged on MP subsequently underwent tapering and discontinuation of their prednisone dose.

In summary, for the vast majority of kidney transplant recipients, multiple studies have shown no disadvantage to RDP. Moreover, when compared to low‐dose MP, RDP has fewer prednisone‐related side effects. However, there is limited data on long‐term outcomes [30, 31]. Our current analysis shows that at 20 years posttransplant, RDP compared with MP, continues to be associated with better or equivalent patient, graft, and death‐censored graft survival.

## Conflicts of Interest

The authors declare no conflicts of interest.

## Supporting information




**Figure S1**. Adjusted patient (A), graft (B) and death−censored graft (C) survival for deceased donor first transplants treated with rapid discontinuation of prednisone (RDP) compared with maintenance prednisone (MP).
**Figure S2**. Adjusted patient (A), graft (B) and death−censored graft (C) survival for deceased donor second transplants treated with rapid discontinuation of prednisone (RDP) compared with maintenance prednisone (MP).
**Figure S3**. Adjusted patient (A), graft (B) and death−censored graft (C) survival for living donor first transplants treated with rapid discontinuation of prednisone (RDP) compared with maintenance prednisone (MP).
**Figure S4**. Adjusted patient (A), graft (B) and death−censored graft (C) survival for living donor second transplants treated with rapid discontinuation of prednisone (RDP) compared with maintenance prednisone (MP).

**Table S1**. Donor and recipient characteristics of those treated with rapid discontinuation of prednisone (RDP) compared with maintenance prednisone (MP) among deceased donor, first transplant recipients. Continuous variables were summarized by median [interquartile range] and categorical variables were summarized by frequency (percent).
**Table S2**. Donor and recipient characteristics of those treated with rapid discontinuation of prednisone (RDP) compared with maintenance prednisone (MP) among deceased donor, second transplant recipients. Continuous variables were summarized by median [interquartile range] and categorical variables were summarized by frequency (percent).
**Table S3**. Donor and recipient characteristics of those treated with rapid discontinuation of prednisone (RDP) compared with maintenance prednisone (MP) among living donor, first transplant recipients. Continuous variables were summarized by median [interquartile range] and categorical variables were summarized by frequency (percent).
**Table S4**. Donor and recipient characteristics of those treated with rapid discontinuation of prednisone (RDP) compared with maintenance prednisone (MP) among living donor, second transplant recipients. Continuous variables were summarized by median [interquartile range] and categorical variables were summarized by frequency (percent).

## Data Availability

The data that support the findings of this study are available from the corresponding author upon reasonable request. The data are not publicly available due to privacy or ethical restrictions.
