# Supplementary material for: Twenty‐Year Kidney Transplant Outcomes With Prednisone‐Free Maintenance Immunosuppression: A Matched Control Analysis
Source: Clin Transplant. 2025 Oct 14;39(10):e70297. doi: 10.1111/ctr.70297 (PMC12520163; doi:10.1111/ctr.70297)
Supplement: Supplementary file 2 — Table S1. Donor and recipient characteristics of those treated with rapid discontinuation of prednisone (RDP) compared with maintenance prednisone (MP) among deceased donor, first transplant recipients. Continuous variables were summarized by median [interquartile range] and categorical variables were summarized by frequency (percent). Table S2. Donor and recipient characteristics of those treated with rapid discontinuation of prednisone (RDP) compared with maintenance prednisone (MP) among deceased donor, second transplant recipients. Continuous variables were summarized by median [interquartile range] and categorical variables were summarized by frequency (percent). Table S3. Donor and recipient characteristics of those treated with rapid discontinuation of prednisone (RDP) compared with maintenance prednisone (MP) among living donor, first transplant recipients. Continuous variables were summarized by median [interquartile range] and categorical variables were summarized by frequency (percent). Table S4. Donor and recipient characteristics of those treated with rapid discontinuation of prednisone (RDP) compared with maintenance prednisone (MP) among living donor, second transplant recipients. Continuous variables were summarized by median [interquartile range] and categorical variables were summarized by frequency (percent). [file CTR-39-e70297-s001.docx]

**Supplement**

**Table S1:** Donor and recipient characteristics of those treated with rapid discontinuation of prednisone (RDP) compared with maintenance prednisone (MP) among deceased donor, first transplant recipients. Continuous variables were summarized by median [interquartile range] and categorical variables were summarized by frequency (percent).

|  | **level** | **MP** | **RDP** | **SMD** |
| --- | --- | --- | --- | --- |
| **n** |  | 1485 | 297 |  |
| **Recipient Age at Transplant (years) (IQR)** |  | 53.8 [44.1, 62.3] | 55.7 [44.8, 64.3] | 0.011 |
| **Recipient Race** | Not Black or African American | 1369 (92.2) | 275 (92.6) | 0.015 |
|  | Black or African American | 116 (7.8) | 22 (7.4) |  |
| **Recipient Sex** | Female | 682 (45.9) | 134 (45.1) | 0.016 |
|  | Male | 803 (54.1) | 163 (54.9) |  |
| **Recipient Primary Disease** | Type 1 Diabetes | 163 (11.0) | 39 (13.1) | 0.096 |
|  | Type 2 Diabetes | 319 (21.5) | 60 (20.2) |  |
|  | Focal Segmental Glomerulosclerosis | 87 (5.9) | 16 (5.4) |  |
|  | Hypertension | 191 (12.9) | 44 (14.8) |  |
|  | IgA nephropathy | 76 (5.1) | 14 (4.7) |  |
|  | Other | 487 (32.8) | 94 (31.6) |  |
|  | Polycystic Kidney Disease | 162 (10.9) | 30 (10.1) |  |
| **Transplant Year** |  | 2006.5 [2004.0, 2008.4] | 2006.5 [2004.2, 2008.1] | 0.009 |
| **Donor Age (years)** |  | 43.0 [25.0, 53.0] | 42.0 [23.0, 54.0] | 0.023 |
| **Donor Race** | Not Black or African American | 1415 (95.3) | 281 (94.6) | 0.031 |
|  | Black or African American | 70 (4.7) | 16 (5.4) |  |
| **Most Recent Peak PRA** |  | 0.0 [0.0, 27.0] | 0.0 [0.0, 20.0] | 0.064 |
| **Donor Sex** | Female | 570 (38.4) | 118 (39.7) | 0.028 |
|  | Male | 915 (61.6) | 179 (60.3) |  |
| **Induction Immunosuppression** | Campath | 11 (0.7) | 4 (1.3) | 0.067 |
|  | Interleukin-2 | 17 (1.1) | 3 (1.0) |  |
|  | None | 30 (2.0) | 5 (1.7) |  |
|  | Other Induction Regimen | 79 (5.3) | 15 (5.1) |  |
|  | Thymoglobulin | 1348 (90.8) | 270 (90.9) |  |
| **Number of HLA Mismatches** | 0 | 302 (20.3) | 66 (22.2) | 0.082 |
|  | 1 | 19 (1.3) | 4 (1.3) |  |
|  | 2 | 101 (6.8) | 24 (8.1) |  |
|  | 3 | 215 (14.5) | 43 (14.5) |  |
|  | 4 | 335 (22.6) | 62 (20.9) |  |
|  | 5 | 328 (22.1) | 65 (21.9) |  |
|  | 6 | 185 (12.5) | 33 (11.1) |  |
| **Received Previous Kidney** | No | 1485 (100.0) | 297 (100.0) | <0.001 |
|  | Yes | 0 (0.0) | 0 (0.0) |  |
| **Recipient Diabetes Diagnosis** | No | 945 (63.6) | 186 (62.6) | 0.021 |
|  | Yes | 540 (36.4) | 111 (37.4) |  |
| **Pre-emptive Transplant** | No | 1222 (82.3) | 236 (79.5) | 0.072 |
|  | Yes | 263 (17.7) | 61 (20.5) |  |
| **Recipient BMI (kg/m2)** |  | 27.4 [23.9, 31.7] | 27.6 [23.8, 31.8] | 0.026 |
| **Recipient Functional Status** | No Assistance | 1147 (77.2) | 228 (76.8) | 0.065 |
|  | Some Assistance | 324 (21.8) | 64 (21.5) |  |
|  | Total Assistance | 14 (0.9) | 5 (1.7) |  |
| **Donor Hypertension** | No | 1183 (79.7) | 242 (81.5) | 0.046 |
|  | Yes | 302 (20.3) | 55 (18.5) |  |
| **Donor Creatinine (mg/dL)** |  | 0.9 [0.7, 1.2] | 0.9 [0.7, 1.2] | 0.056 |
| **Cold Ischemic Time (hours)** |  | 14.1 [9.0, 19.7] | 13.8 [10.2, 19.0] | 0.039 |
| **Donor Cause of Death** | Other | 680 (45.8) | 137 (46.1) | 0.041 |
|  | Anoxia | 236 (15.9) | 51 (17.2) |  |
|  | Stroke | 569 (38.3) | 109 (36.7) |  |
| **Maintenance Immunosuppression** | Cyclosporine Mycophenolate Mofetil | 144 (9.7) | 195 (65.7) | 1.605 |
|  | Other Maintenance Regimen | 234 (15.8) | 17 (5.7) |  |
|  | Tacrolimus Mycophenolate Mofetil | 1083 (72.9) | 63 (21.2) |  |
|  | Tacrolimus mTOR | 24 (1.6) | 22 (7.4) |  |

**Table S2:** Donor and recipient characteristics of those treated with rapid discontinuation of prednisone (RDP) compared with maintenance prednisone (MP) among deceased donor, second transplant recipients. Continuous variables were summarized by median [interquartile range] and categorical variables were summarized by frequency (percent).

|  | **level** | **MP** | **RDP** | **SMD** |
| --- | --- | --- | --- | --- |
| **n** |  | 320 | 64 |  |
| **Recipient Age at Transplant (years) IQR)** |  | 45.9 [36.6, 53.9] | 43.0 [29.9, 56.7] | 0.111 |
| **Recipient Race** | Not Black or African American | 299 (93.4) | 59 (92.2) | 0.048 |
|  | Black or African American | 21 (6.6) | 5 (7.8) |  |
| **Recipient Sex** | Female | 145 (45.3) | 31 (48.4) | 0.063 |
|  | Male | 175 (54.7) | 33 (51.6) |  |
| **Recipient Primary Disease** | Type 1 Diabetes | 20 (6.2) | 4 (6.2) | 0.474 |
|  | Type 2 Diabetes | 11 (3.4) | 1 (1.6) |  |
|  | Focal Segmental Glomerulosclerosis | 15 (4.7) | 6 (9.4) |  |
|  | Hypertension | 27 (8.4) | 1 (1.6) |  |
|  | IgA nephropathy | 16 (5.0) | 1 (1.6) |  |
|  | Other | 217 (67.8) | 45 (70.3) |  |
|  | Polycystic Kidney Disease | 14 (4.4) | 6 (9.4) |  |
| **Transplant Year** |  | 2006.6 [2003.9, 2008.3] | 2006.6 [2004.3, 2008.6] | 0.117 |
| **Donor Age (years)** |  | 38.0 [23.0, 49.2] | 39.0 [22.0, 47.0] | 0.001 |
| **Donor Race** | Not Black or African American | 307 (95.9) | 60 (93.8) | 0.099 |
|  | Black or African American | 13 (4.1) | 4 (6.2) |  |
| **Most Recent Peak PRA** |  | 53.5 [11.0, 91.0] | 85.0 [21.0, 98.0] | 0.300 |
| **Donor Sex** | Female | 141 (44.1) | 23 (35.9) | 0.166 |
|  | Male | 179 (55.9) | 41 (64.1) |  |
| **Induction Immunosuppression** | Campath | 1 (0.3) | 0 (0.0) | 0.223 |
|  | Interleukin-2 | 3 (0.9) | 0 (0.0) |  |
|  | None | 1 (0.3) | 1 (1.6) |  |
|  | Other Induction Regimen | 9 (2.8) | 1 (1.6) |  |
|  | Thymoglobulin | 306 (95.6) | 62 (96.9) |  |
| **Number of HLA Mismatches** | 0 | 93 (29.1) | 18 (28.1) | 0.230 |
|  | 1 | 2 (0.6) | 0 (0.0) |  |
|  | 2 | 32 (10.0) | 5 (7.8) |  |
|  | 3 | 35 (10.9) | 11 (17.2) |  |
|  | 4 | 70 (21.9) | 14 (21.9) |  |
|  | 5 | 60 (18.8) | 10 (15.6) |  |
|  | 6 | 28 (8.8) | 6 (9.4) |  |
| **Received Previous Kidney** | No | 0 (0.0) | 0 (0.0) | <0.001 |
|  | Yes | 320 (100.0) | 64 (100.0) |  |
| **Recipient Diabetes Diagnosis** | No | 269 (84.1) | 57 (89.1) | 0.147 |
|  | Yes | 51 (15.9) | 7 (10.9) |  |
| **Pre-emptive Transplant** | No | 268 (83.8) | 61 (95.3) | 0.385 |
|  | Yes | 52 (16.2) | 3 (4.7) |  |
| **Recipient BMI (kg/m2)** |  | 25.1 [22.1, 29.0] | 24.5 [21.3, 29.7] | 0.026 |
| **Recipient Functional Status** | No Assistance | 230 (71.9) | 47 (73.4) | 0.178 |
|  | Some Assistance | 85 (26.6) | 17 (26.6) |  |
|  | Total Assistance | 5 (1.6) | 0 (0.0) |  |
| **Donor Hypertension** | No | 276 (86.2) | 51 (79.7) | 0.175 |
|  | Yes | 44 (13.8) | 13 (20.3) |  |
| **Donor Creatinine (mg/dL)** |  | 0.9 [0.7, 1.2] | 1.0 [0.8, 1.3] | 0.193 |
| **Cold Ischemic Time (hours)** |  | 16.2 [10.7, 21.5] | 14.7 [10.2, 18.2] | 0.239 |
| **Donor Cause of Death** | Other | 153 (47.8) | 30 (46.9) | 0.049 |
|  | Anoxia | 59 (18.4) | 11 (17.2) |  |
|  | Stroke | 108 (33.8) | 23 (35.9) |  |
| **Maintenance Immunosuppression** | Cyclosporine Mycophenolate Mofetil | 23 (7.2) | 39 (60.9) | 1.667 |
|  | Other Maintenance Regimen | 46 (14.4) | 3 (4.7) |  |
|  | Tacrolimus Mycophenolate Mofetil | 245 (76.6) | 15 (23.4) |  |
|  | Tacrolimus mTOR | 6 (1.9) | 7 (10.9) |  |

**Table S3:** Donor and recipient characteristics of those treated with rapid discontinuation of prednisone (RDP) compared with maintenance prednisone (MP) among living donor, first transplant recipients. Continuous variables were summarized by median [interquartile range] and categorical variables were summarized by frequency (percent).

|  | **level** | **MP** | **RDP** | **SMD** |
| --- | --- | --- | --- | --- |
| **n** |  | 2094 | 698 |  |
| **Recipient Age at Transplant (years) (IQR)** |  | 47.0 [34.6, 56.8] | 47.0 [35.5, 55.9] | 0.039 |
| **Recipient Race** | Not Black or African American | 2023 (96.6) | 673 (96.4) | 0.010 |
|  | Black or African American | 71 (3.4) | 25 (3.6) |  |
| **Recipient Sex** | Female | 773 (36.9) | 258 (37.0) | 0.001 |
|  | Male | 1321 (63.1) | 440 (63.0) |  |
| **Recipient Primary Disease** | Type 1 Diabetes | 230 (11.0) | 157 (22.5) | 0.315 |
|  | Type 2 Diabetes | 256 (12.2) | 72 (10.3) |  |
|  | Focal Segmental Glomerulosclerosis | 159 (7.6) | 44 (6.3) |  |
|  | Hypertension | 182 (8.7) | 57 (8.2) |  |
|  | IgA nephropathy | 184 (8.8) | 56 (8.0) |  |
|  | Other | 779 (37.2) | 219 (31.4) |  |
|  | Polycystic Kidney Disease | 304 (14.5) | 93 (13.3) |  |
| **Recipient Transplant Year** |  | 2005.5 [2003.3, 2007.9] | 2005.7 [2003.7, 2007.5] | 0.022 |
| **Donor Age (years)** |  | 41.0 [33.0, 49.0] | 43.0 [35.0, 49.0] | 0.023 |
| **Donor Race** | Not Black or African American | 2026 (96.8) | 677 (97.0) | 0.014 |
|  | Black or African American | 68 (3.2) | 21 (3.0) |  |
| **Most Recent Peak PRA** |  | 0.0 [0.0, 4.0] | 0.0 [0.0, 3.0] | 0.101 |
| **Donor Sex** | Female | 1267 (60.5) | 431 (61.7) | 0.025 |
|  | Male | 827 (39.5) | 267 (38.3) |  |
| **Induction Immunosuppression** | Campath | 2 (0.1) | 1 (0.1) | 0.022 |
|  | Interleukin-2 | 10 (0.5) | 3 (0.4) |  |
|  | None | 113 (5.4) | 38 (5.4) |  |
|  | Other Induction Regimen | 46 (2.2) | 17 (2.4) |  |
|  | Thymoglobulin | 1923 (91.8) | 639 (91.5) |  |
| **Number of HLA MM** | 0 | 153 (7.3) | 63 (9.0) | 0.070 |
|  | 1 | 113 (5.4) | 36 (5.2) |  |
|  | 2 | 366 (17.5) | 114 (16.3) |  |
|  | 3 | 587 (28.0) | 194 (27.8) |  |
|  | 4 | 306 (14.6) | 98 (14.0) |  |
|  | 5 | 374 (17.9) | 128 (18.3) |  |
|  | 6 | 195 (9.3) | 65 (9.3) |  |
| **Received Previous Kidney** | No | 2094 (100.0) | 698 (100.0) | <0.001 |
|  | Yes | 0 (0.0) | 0 (0.0) |  |
| **Recipient Diabetes Diagnosis** | No | 1553 (74.2) | 453 (64.9) | 0.202 |
|  | Yes | 541 (25.8) | 245 (35.1) |  |
| **Pre-emptive Transplant** | No | 1255 (59.9) | 382 (54.7) | 0.105 |
|  | Yes | 839 (40.1) | 316 (45.3) |  |
| **Recipient BMI (kg/m2)** |  | 26.0 [22.6, 30.2] | 26.1 [22.4, 30.2] | 0.034 |
| **Recipient Functional Status** | No Assistance | 1815 (86.7) | 603 (86.4) | 0.031 |
|  | Some Assistance | 264 (12.6) | 88 (12.6) |  |
|  | Total Assistance | 15 (0.7) | 7 (1.0) |  |
| **Donor Hypertension** | No | 2071 (98.9) | 677 (97.0) | 0.135 |
|  | Yes | 23 (1.1) | 21 (3.0) |  |
| **Donor Creatinine (mg/dL)** |  | 0.9 [0.8, 1.0] | 0.9 [0.8, 1.0] | 0.043 |
| **Maintenance Immunosuppression** | Cyclosporine Mycophenolate Mofetil | 220 (10.5) | 430 (61.6) | 1.593 |
|  | Other Maintenance Regimen | 387 (18.5) | 29 (4.2) |  |
|  | Tacrolimus Mycophenolate Mofetil | 1449 (69.2) | 153 (21.9) |  |
|  | Tacrolimus mTOR | 38 (1.8) | 86 (12.3) |  |

**Table S4:** Donor and recipient characteristics of those treated with rapid discontinuation of prednisone (RDP) compared with maintenance prednisone (MP) among living donor, second transplant recipients. Continuous variables were summarized by median [interquartile range] and categorical variables were summarized by frequency (percent).

|  | **level** | **MP** | **RDP** | **SMD** |
| --- | --- | --- | --- | --- |
| **n** |  | 195 | 65 |  |
| **Recipient Age at Transplant (years) (IQR)** |  | 42.5 [32.2, 50.2] | 40.8 [26.2, 49.8] | 0.160 |
| **Recipient Race** | Not Black or African American | 191 (97.9) | 64 (98.5) | 0.039 |
|  | Black or African American | 4 (2.1) | 1 (1.5) |  |
| **Recipient Sex** | Female | 75 (38.5) | 22 (33.8) | 0.096 |
|  | Male | 120 (61.5) | 43 (66.2) |  |
| **Recipient Primary Disease** | Type 1 Diabetes | 20 (10.3) | 9 (13.8) | 0.495 |
|  | Type 2 Diabetes | 3 (1.5) | 0 (0.0) |  |
|  | Focal Segmental Glomerulosclerosis | 12 (6.2) | 5 (7.7) |  |
|  | Hypertension | 24 (12.3) | 2 (3.1) |  |
|  | IgA nephropathy | 9 (4.6) | 6 (9.2) |  |
|  | Other | 112 (57.4) | 41 (63.1) |  |
|  | Polycystic Kidney Disease | 15 (7.7) | 2 (3.1) |  |
| **Recipient Transplant Year** |  | 2005.4 [2003.5, 2007.8] | 2005.8 [2004.5, 2007.6] | 0.145 |
| **Donor Age** |  | 40.0 [29.5, 48.0] | 38.0 [30.0, 46.0] | 0.042 |
| **Donor Race** | Not Black or African American | 192 (98.5) | 64 (98.5) | <0.001 |
|  | Black or African American | 3 (1.5) | 1 (1.5) |  |
| **Most Recent Peak PRA** |  | 10.0 [0.0, 58.0] | 33.0 [3.0, 84.0] | 0.394 |
| **Donor Sex** | Female | 113 (57.9) | 34 (52.3) | 0.114 |
|  | Male | 82 (42.1) | 31 (47.7) |  |
| **Induction Immunosuppression** | Campath | 0 (0.0) | 0 (0.0) | 0.049 |
|  | Interleukin-2 | 0 (0.0) | 0 (0.0) |  |
|  | None | 5 (2.6) | 2 (3.1) |  |
|  | Other Induction Regimen | 4 (2.1) | 1 (1.5) |  |
|  | Thymoglobulin | 186 (95.4) | 62 (95.4) |  |
| **Number of HLA MM** | 0 | 26 (13.3) | 9 (13.8) | 0.218 |
|  | 1 | 6 (3.1) | 2 (3.1) |  |
|  | 2 | 30 (15.4) | 13 (20.0) |  |
|  | 3 | 55 (28.2) | 20 (30.8) |  |
|  | 4 | 22 (11.3) | 8 (12.3) |  |
|  | 5 | 38 (19.5) | 9 (13.8) |  |
|  | 6 | 18 (9.2) | 4 (6.2) |  |
| **Received Previous Kidney** | No | 0 (0.0) | 0 (0.0) | <0.001 |
|  | Yes | 195 (100.0) | 65 (100.0) |  |
| **Recipient Diabetes Diagnosis** | No | 163 (83.6) | 54 (83.1) | 0.014 |
|  | Yes | 32 (16.4) | 11 (16.9) |  |
| **Pre-emptive Transplant** | No | 126 (64.6) | 56 (86.2) | 0.516 |
|  | Yes | 69 (35.4) | 9 (13.8) |  |
| **Recipient BMI (kg/m2)** |  | 24.0 [21.4, 27.9] | 23.4 [20.8, 27.2] | 0.144 |
| **Recipient Functional Status** | No Assistance | 156 (80.0) | 55 (84.6) | 0.121 |
|  | Some Assistance | 39 (20.0) | 10 (15.4) |  |
|  | Total Assistance | 0 (0.0) | 0 (0.0) |  |
| **Donor Hypertension** | No | 193 (99.0) | 63 (96.9) | 0.145 |
|  | Yes | 2 (1.0) | 2 (3.1) |  |
| **Donor Creatinine (mg/dL)** |  | 0.9 [0.7, 1.0] | 0.9 [0.8, 1.0] | 0.022 |
| **Maintenance Immunosuppression** | Cyclosporine Mycophenolate Mofetil | 14 (7.2) | 35 (53.8) | 1.416 |
|  | Other Maintenance Regimen | 28 (14.4) | 2 (3.1) |  |
|  | Tacrolimus Mycophenolate Mofetil | 149 (76.4) | 21 (32.3) |  |
|  | Tacrolimus mTOR | 4 (2.1) | 7 (10.8) |  |
