# Supplementary material for: Twenty‐Year Kidney Transplant Outcomes With Prednisone‐Free Maintenance Immunosuppression: A Matched Control Analysis
Source: Clin Transplant. 2025 Oct 14;39(10):e70297. doi: 10.1111/ctr.70297 (PMC12520163; doi:10.1111/ctr.70297)

Figure S1.

Adjusted patient (A), graft (B) and death−censored graft (C) survival for deceased donor first transplants treated with rapid discontinuation of prednisone (RDP) compared with maintenance prednisone (MP)

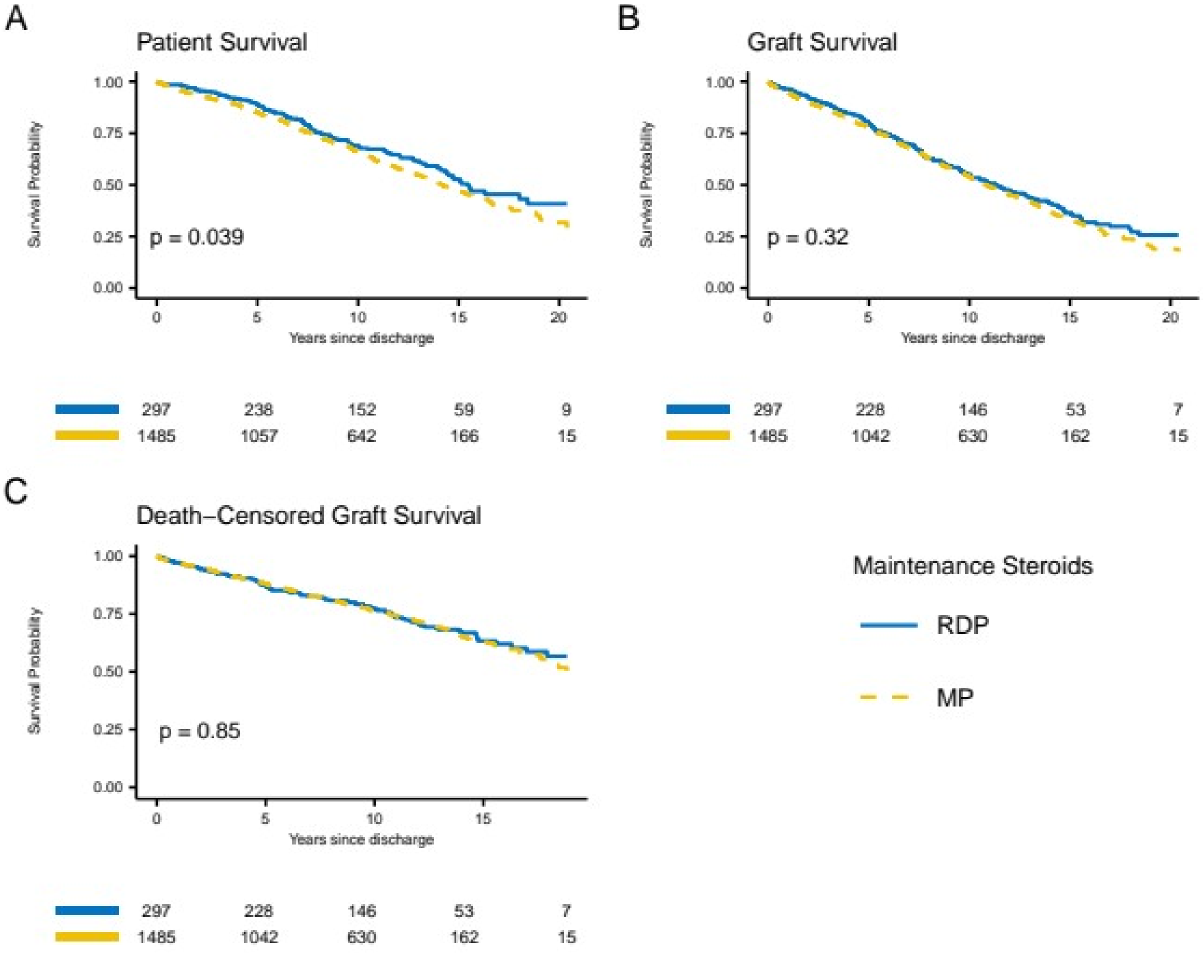

Figure S2.

Adjusted patient (A), graft (B) and death−censored graft (C) survival for deceased donor second transplants treated with rapid discontinuation of prednisone (RDP) compared with maintenance prednisone (MP)

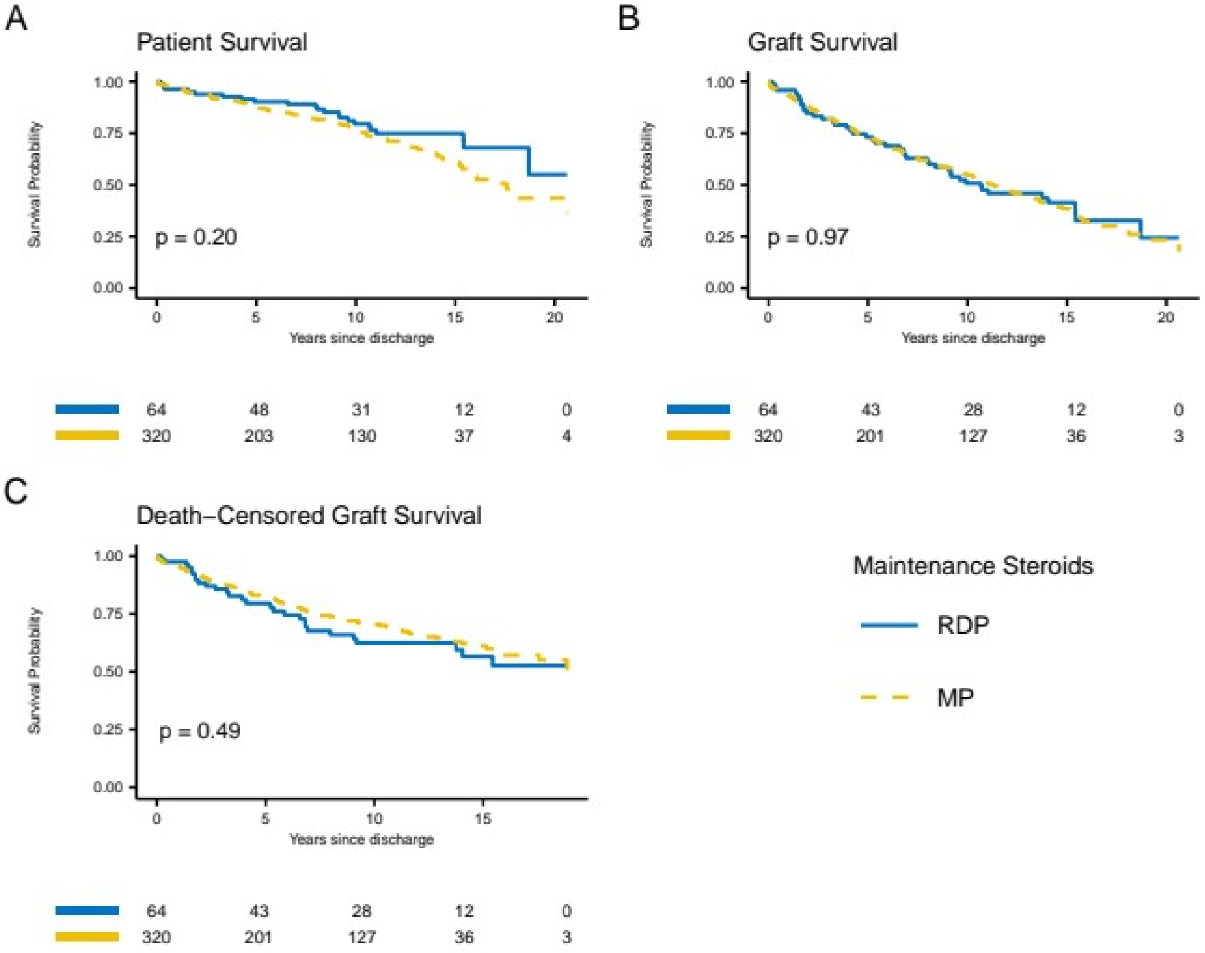

Figure S3.

Adjusted patient (A), graft (B) and death−censored graft (C) survival for living donor first transplants treated with rapid discontinuation of prednisone (RDP) compared with maintenance prednisone (MP)

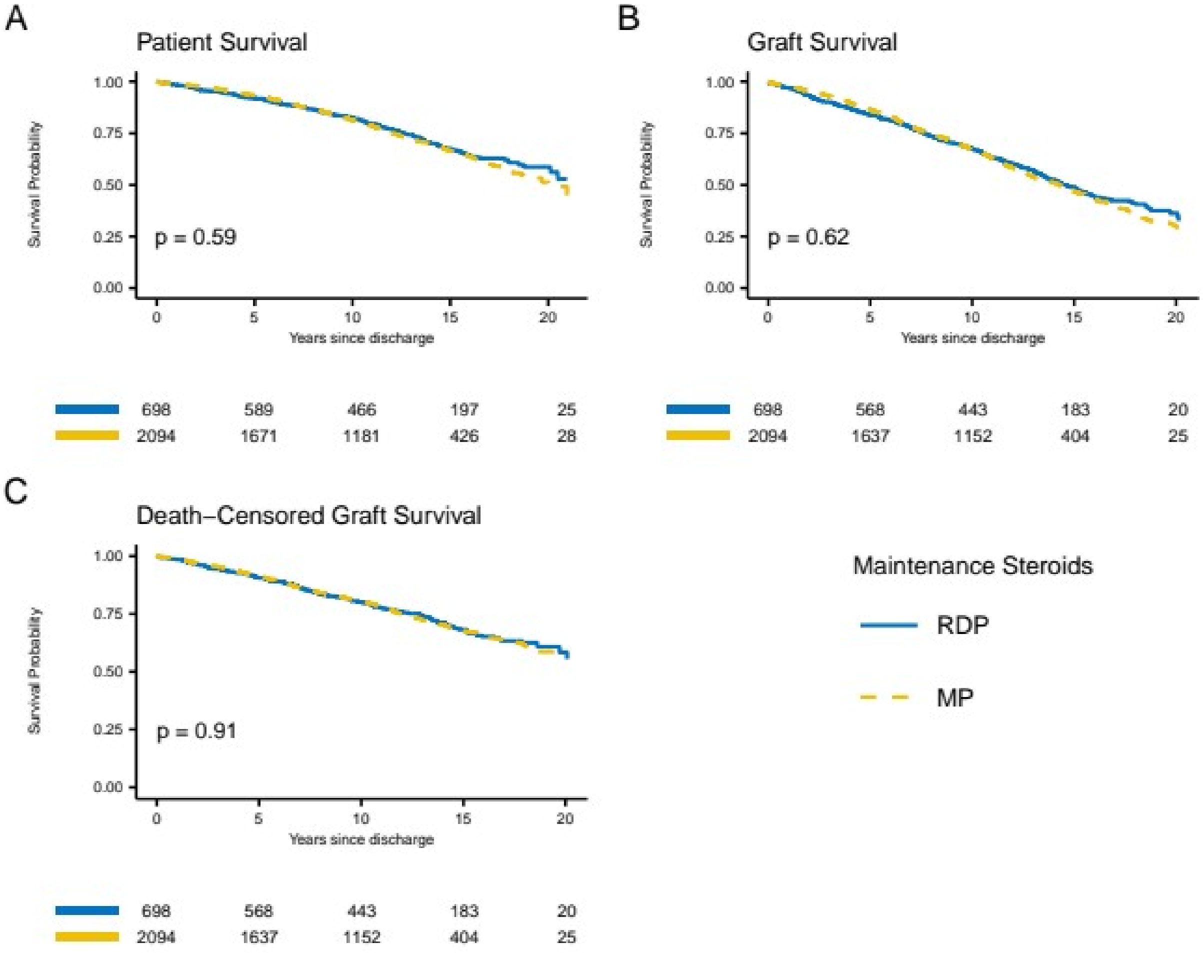

Figure S4.

Adjusted patient (A), graft (B) and death−censored graft (C) survival for living donor second transplants treated with rapid discontinuation of prednisone (RDP) compared with maintenance prednisone (MP)

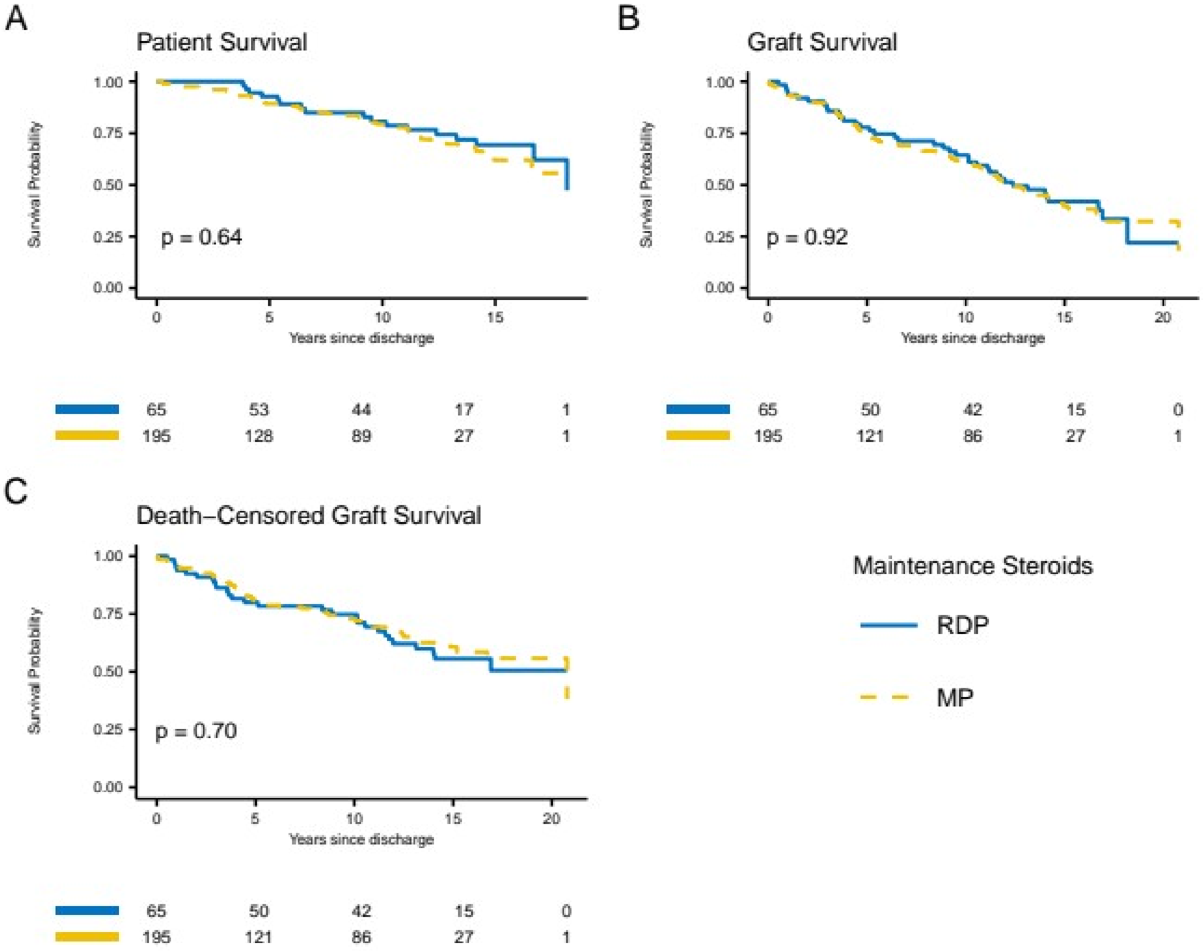

Supplement: Supplementary file 2 [file CTR-39-e70297-s002.docx]
